# Adherence to Lifestyle Recommendations for Bone Health in Older Adults with and without Osteoporosis: Cross-Sectional Results of the OUTDOOR ACTIVE Study

**DOI:** 10.3390/nu14122463

**Published:** 2022-06-14

**Authors:** Birte Marie Albrecht, Imke Stalling, Linda Foettinger, Carina Recke, Karin Bammann

**Affiliations:** Institute for Public Health and Nursing Research (IPP), University of Bremen, 28359 Bremen, Germany; stalling@uni-bremen.de (I.S.); foettili@uni-bremen.de (L.F.); c.recke@uni-bremen.de (C.R.); bammann@uni-bremen.de (K.B.)

**Keywords:** bone health, lifestyle, osteoporosis, preventive behavior

## Abstract

Sustaining good bone health into older age is key for preventing osteoporosis. Bone health is associated with several lifestyle factors. This study investigates older adults’ adherence to bone health-promoting lifestyle recommendations dependent on osteoporosis status. Cross-sectional data of 1610 community-dwelling older adults (65–75 years) residing in Bremen, Germany (53.4% female) were included. The Osteoporosis Self-Assessment Tool and self-reported osteoporosis diagnosis were used to classify participants by osteoporosis status (low risk, high risk, diagnosis). Adherence to bone health recommendations regarding calcium and vitamin D intake, sun exposure, alcohol consumption, resistance/weight-bearing exercise, and physical activity were assessed. Descriptive statistics were applied, stratified by sex and osteoporosis status. A total of 91 women (10.6%) and 15 men (2.0%) reported an osteoporosis diagnosis, 457 women (43.2%) and 311 men (41.4%) were classified as having a high risk, and 311 women (36.2%) and 425 men (56.6%) as having a low risk. Adherence to bone health recommendations was high for calcium intake (93.3–100.0%), vitamin D intake (77.8–93.3%), and sun exposure (86.7–97.7%). Lower adherence was observed regarding resistance/weight-bearing exercise (36.3–54.4%), physical activity (14.3–57.7%), and alcohol consumption (40.0–72.4%). In conclusion, tailored prevention strategies are needed that focus on older adults with an osteoporosis diagnosis or who are at high risk.

## 1. Introduction

Osteoporosis—the most common bone disease [1]—is characterized by low bone mass and a microarchitectural deterioration of bone tissue [2], which lead to a higher risk for fractures [3]. Osteoporotic fractures are not only associated with pain and functional limitations [4], but also with a significant increase in mortality [5]. The prevalence of osteoporosis increases with age, especially after the age of 65 years [6]. Due to hormonal changes in menopause, women are more often affected by osteoporosis [7]. In Germany, the 12-month prevalence of osteoporosis in adults aged 65 years and older is 24.0% in women and 5.6% in men [6]. As the population ages due to demographic change, the prevention of osteoporosis and osteoporotic fractures is becoming more and more relevant.

Sustaining good bone health throughout the lifespan is important for preventing the development of osteoporosis [8,9,10]. However, even after a diagnosis of osteoporosis, a lifestyle promoting bone health can lower the risk of fractures [11]. Key lifestyle choices that are associated with bone health are nutrition, alcohol consumption, smoking, and physical activity [11,12,13]. Calcium and vitamin D play a central role in bone metabolism [14]. Foods rich in calcium include dairy products, green vegetables, and mineral water [8,15]. Vitamin D is contained in higher amounts in, e.g., fat fish and eggs [16], however, the body’s own production of vitamin D through sun exposure is vital for a sufficient supply [17]. For this, the International Osteoporosis Foundation recommends spending at least 15 min per day outside [18]. Excessive alcohol consumption (over one glass per day in women or over two glasses per day in men) is associated with a decrease in bone mass and strength due to a bone remodeling imbalance and should, therefore, be avoided [19]. Smoking has similar effects on the skeletal system and should be ceased [20]. Lastly, physical activity has an impact on bone health. An overall active lifestyle according to the physical activity guidelines of the World Health Organization (WHO) is recommended [21,22]. Moreover, a focus on weight-bearing exercises (e.g., hiking, dancing) and resistance training is important as these can help to build and maintain bone strength [18,21,23].

The preventive behavior for osteoporosis has not been studied extensively. Knowledge of osteoporosis—as one proposed influential factor for preventive behavior [24]—appears to be insufficient worldwide [25,26]. Von Hurst et al. observed a small increase in knowledge with age in women, however, perceptions of personal susceptibility and seriousness remained on a low level [27]. Other studies also report that older adults are not personally concerned by osteoporosis and underestimate the seriousness of this disease [26,28]. This is reflected in their preventive behavior. Researchers from Taiwan reported a high prevalence of adults with osteoporosis who did not exercise on a regular basis and did not have an adequate intake of calcium and vitamin D [29]. Vitamin D insufficiency is also very common across all age groups in Germany [30,31].

Investigating the osteoporosis preventive behavior of older adults can provide meaningful insights for tailoring prevention approaches to specifically address potential gaps. Therefore, the objective of this study is to assess older adults’ adherence to lifestyle recommendations related to bone health dependent on the osteoporosis status. 

## 2. Materials and Methods

### 2.1. Study Design and Population

This study is based on pooled data from the baseline survey of the OUTDOOR ACTIVE pilot study (OA1; 02/2015-01/2018) and the OUTDOOR ACTIVE cluster-randomized controlled trial (OA2; 02/2018-12/2022), which both aimed to develop and implement an outdoor physical activity promotion program for older adults by applying a participatory approach. In OA1, this approach was developed and implemented in the five subdistricts of Hemelingen in Bremen, Germany. In the second funding phase (OA2), the developed approach was tested in eight randomly selected subdistricts (four intervention, four control) of Bremen, Germany [32]. OUTDOOR ACTIVE is embedded in the prevention research network AEQUIPA, which centers on investigating physical activity as a key factor for healthy ageing from different perspectives and is located in the northwest of Germany [33].

Address data for all adults in the age group 65–75 years living in one of the selected subdistricts in OA1 and OA2 were obtained from the registry office. From this list, individuals living in care facilities were excluded. The remaining received a letter including a questionnaire on influential factors of physical activity and an invitation to participate in a health assessment. The health assessment included the measurement of body height, body weight, waist circumference, and blood pressure. Moreover, several physical fitness dimensions were assessed with an adapted version [34] of the Senior Fitness Test developed by Rikli and Jones [35]. The baseline survey was complemented by a physical activity measurement applying accelerometry over seven days.

In total, 11,079 individuals were registered in the selected study regions (OA1: *n* = 4226; OA2: *n* = 6853). Of those, 461 could not participate due to acute health problems (OA1: *n* = 242; OA2: *n* = 219) and 125 deceased (OA1: *n* = 56; OA2: *n* = 69), 450 individuals moved outside the study region (OA1: *n* = 295; OA2: *n* = 155), and 77 could not participate because of language barriers (OA1: *n* = 22; OA2: *n* = 55). Of the remaining 9966 confirmed eligible individuals (OA1: *n* = 3611; OA2: *n* = 6355), 3425 were never reached (OA1: *n* = 720; OA2: *n* = 2705), and 4247 refused to participate (OA1: *n* = 2052; OA2: *n* = 2195). Furthermore, 151 individuals in one subdistrict were never contacted because the end of the survey period in this subdistrict was reached and the actual sample size of the subdistrict already exceeded the calculated sample size. Overall, 2143 individuals participated in at least one part of the pilot study or the cluster-randomized trial (OA1: *n* = 917; OA2: *n* = 1226; overall response: 21.5%). Only participants who at least filled out the questionnaire and took part in the health assessment were included in the analyses (*n* = 1610; OA1: *n* = 621; OA2: *n* = 989).

All participants provided written informed consent. The pilot study and the cluster-randomized trial were both approved by the ethical committee of the University of Bremen.

### 2.2. Measures 

#### 2.2.1. Osteoporosis Status

Diagnosis of osteoporosis was assessed via questionnaire. The questionnaire asked participants about their chronic diseases with osteoporosis as one response option. It has been shown that the validity of self-reported osteoporosis is moderate-to-good [36]. Osteoporosis risk was assessed for participants without a diagnosis of osteoporosis using the Osteoporosis Self-Assessment Tool (OST = (body weight (in kg) – age (in years)) × 0.2) by Koh et al. [37], which has shown good predictive values for bone mineral density [38]. As the original cut-points for risk classification of the score were developed for a female Asian population, we applied the cut-points suggested by Erjiang et al. for an Irish population (high risk of osteoporosis: ≤0 in women and ≤2 in men) [39]. Age and body weight (Kern MPC 250K100M personal floor scale, Kern & Sohn GmbH, Ballingen, Germany) were assessed during the health assessment. 

#### 2.2.2. Health-Related Variables

Self-rated health, physical functioning, and medication were self-reported in the questionnaire. Self-rated health was assessed with a single question from the Short Form 36 [40]. To determine physical functioning, the Physical Functioning Scale from the Short Form 36 was applied [41], where participants had to rate their level of limitations (not limited, limited a little, limited a lot) for ten activities of daily living. For scoring, the responses were recoded to 100 (not limited), 50 (limited a little), and 0 (limited a lot), and the mean value of all ten items was determined [42]. Participants were asked to list all medications they currently take on a daily basis. These were then coded according to the Anatomical Therapeutic Chemical (ATC) classification [43]. Drugs used for the treatment of bone diseases (ATC code beginning with M05) were included in this study. 

During the health assessment, peak body height was asked and current body height was measured with a Seca 217 mobile stadiometer (Seca GmbH & Co. KG, Hamburg, Germany). Height shrinkage was determined by subtracting the current body height from peak body height. Body mass index was calculated (BMI = body weight (in kg)/body height (in m)^2^) and classified into underweight, normal weight, overweight, and obesity according to the World Health Organization [44]. The adapted version of the Senior Fitness Test [35] included the following measurements: handgrip strength, 30 s chair stand test (lower body strength), 2 min step test (aerobic endurance), sit-and-reach test (lower body flexibility), and back scratch test (upper body flexibility). These are described in more detail elsewhere [34]. Performance in the physical fitness measurements was classified according to previously published sex- and age-specific normative values [34]. For this step, a standard method was applied where individual scores within the second and third quartile are defined as being in norm [35]. Therefore, participants’ scores falling within the first quartile were classified as being under norm. Additionally, balance was assessed using the 4-stage balance test [45].

#### 2.2.3. Preventive Behavior

Dietary intake, medication, alcohol consumption, sun exposure, and participation in resistance/weight-bearing exercise were assessed via questionnaire. Sufficient calcium and vitamin D intake was determined by combining dietary intake and medication. For dietary intake, a self-developed food frequency questionnaire was applied. The food frequency questionnaire was based on the validated questionnaire of the German Health Examination Survey for Adults [46]. The current frequency of consumption (never, once a month or less, 2–3 times a month, once a week, several times a week, (nearly) daily) was assessed for the following foods: hard cheese, soft cheese, milk/buttermilk, yogurt/kefir, eggs, wholegrain products, legumes, cabbage/green vegetables, meat, fat fish, alcohol, and mineral water. Participants who either (1) consumed mineral water daily, or (2) consumed a minimum of two out of five calcium-rich foods (hard cheese, soft cheese, milk/buttermilk, yogurt/kefir, cabbage/green vegetables) at least several times a week, or (3) supplemented calcium (ATC code beginning with A12AA or A12AX) were considered to have sufficient calcium intake. To be categorized as having sufficient vitamin D intake, participants had to either (1) consume hard cheese or eggs daily, or (2) consume hard cheese and eggs several times a week, or (3) consume fat fish once a week, or (4) supplement vitamin D (ATC code beginning with A11CC or A12AX). 

Participants were asked how much time they spend outdoors during the day. The response options were the following: under 5 min, 5 to under 20 min, 20 to under 60 min, 1 h and more. In OA2, this item was assessed separately for summer and winter. To pool these data with OA1 data, the response indicating more time spent outdoors was used. Participants who spent at least 20 min outdoors were considered to fulfill the recommendations for sufficient sun exposure. 

In regard to alcohol consumption, participants were categorized as fulfilling the recommendations if they consumed alcohol a maximum of once a week. 

Participants were asked to list all their regular physical activities and the amount of time (in hours) they engaged in them per week. The activities were then categorized into groups, of which the following were classified as resistance/weight-bearing exercise: hiking/running, gym training, aerobics, sports therapy, dancing, and ball sports. To be categorized as fulfilling the recommendations, participants had to engage in these activities for at least two hours per week.

Total physical activity was objectively assessed via accelerometry. Participants were asked to wear an ActiGraph wGT3X-BT accelerometer (ActiGraph LLC, Pensacola, FL, USA) on their non-dominant wrist for seven consecutive days at day and night. Sampling frequency was set to 30 Hz. Accelerometer data were downloaded and processed using ActiLife (Version 6.13.3, ActiGraph LLC, Pensacola, FL, USA). In a first step, data sets were reintegrated to epoch lengths of 60 s. Non-wear time was excluded based on the algorithm by Troiano et al. [47]. To identify moderate-to-vigorous physical activity (MVPA), the cut-points for the non-dominant wrist published by Bammann et al. were applied [48] with bouts of a minimum of ten minutes and a maximum drop time of two minutes. Processed accelerometer data were exported to SPSS^®^ Statistics version 20.0 (IBM Corp., Armonk, NY, USA). First wear days and days with under ten hours of wear time were excluded. Last wear days were also excluded if the accelerometer was worn for more than eight days. Participants had to have at least four valid wear days to be considered in the analyses. Mean MVPA minutes were calculated by participant and multiplied by seven to estimate weekly MVPA minutes. To fulfill the recommendations, participants had to engage in at least 150 min of MVPA per week.

An overview of the requirements to fulfill the lifestyle requirements for bone health is displayed in Table 1.

#### 2.2.4. Sociodemographic Variables

Information on sex, highest school degree, educational years, net household income, employment history, and living situation (alone/not alone) were assessed through the questionnaire. Highest school degree was categorized into educational status according to the International Standard Classification of Education 1997 (ISCED) [49]. Educational years (school years and training years combined), net household income, and employment history were used to assign each participant a socioeconomic status (for details, see [50]).

### 2.3. Statistical Analyses

Mean OST score of participants without osteoporosis diagnosis and absolute and relative frequencies of osteoporosis status were calculated, stratified by sex. Statistical significance of sex differences in mean OST score and frequency distribution of OG 1–3 were calculated by applying t-test and Pearson’s chi-squared test, respectively. 

For the description of the study population, absolute and relative frequencies for educational status, socioeconomic status, and living situation were determined, stratified by sex and osteoporosis status. Descriptive statistics were calculated for health and physical fitness parameters by sex and osteoporosis status. Mean values were determined for age, height, height shrinkage, weight, and physical functioning. Absolute and relative frequencies are displayed for body mass index, self-rated health, medication intake, handgrip strength, 30 s chair stand test, 2 min step test, sit-and-reach test, back scratch test, and the 4-stage balance test. Absolute and relative frequencies were also calculated for all variables regarding osteoporosis preventive behavior by sex and osteoporosis status. Dependent on educational status, socioeconomic status, and living situation, absolute and relative frequencies were determined for lower adherence to lifestyle recommendations, stratified by sex and osteoporosis status. Statistical significance of differences in OG 1–3 was tested for all variables in women and men, respectively. Analysis of variance was applied for comparing mean values. Pearson’s chi-squared test was used for investigating frequencies. If at least one cell count was below five, Fisher’s exact test was applied. In the case of insufficient storage capacity for Fisher’s exact, Monte Carlo simulation was used as alternative.

All analyses were performed in SPSS^®^ Statistics version 20.0 (IBM Corp., Armonk, NY, USA).

## 3. Results

Table 2 depicts the osteoporosis status of the 1610 participants included in the analyses. In total, 91 women (10.6%) and 15 men (2.0%) reported a diagnosis of osteoporosis (OG 3). In participants without an osteoporosis diagnosis, women had a statistically significantly lower mean OST score compared to men (women: 0.23 ± 2.63 vs. men: 3.25 ± 2.76; *p* < 0.001). According to the OST score, 311 women (36.2%) were classified as having a low risk (OG1) and 457 (53.2%) were classified as having a high risk for osteoporosis (OG 2). In comparison, 425 men (56.6%) were in the low-risk group and 311 (41.4%) in the group with a high risk of osteoporosis. Sex differences regarding the frequency distribution of OG 1–3 were statistically significant (*p* < 0.001).

In Table 3, participants’ characteristics by sex and osteoporosis status are displayed. Women in OG 2 were more often upper class (21.3%) compared to women in OG 1 (14.8%) and OG 3 (11.2%; *p* = 0.018). Regarding educational status and living situation, there were no statistically significant differences by OG. In men, none of the characteristics showed significant group differences. 

The highest mean shrinkage in body height was observed in participants in OG 3 (women: −3.8 ± 2.5 cm, men: −2.7 ± 2.1 cm) (see Table 4). Participants in OG 2 had the lowest mean body weight (women: 62.5 ± 6.4 kg, men: 74.6 ± 6.1 kg). This is also reflected in the classification of the body mass index. A 33.9% proportion of women, and 46.3% of men, in OG 2 were overweight or obese, while this was the case for 93.9% of women and 92.2% of men in OG1 and 41.8% of women and 66.7% of men in OG 3. Across all osteoporosis groups, men (OG 1: 26.3%, OG 2: 36.5%, OG 3: 30.8%) reported very good or excellent self-rated health more often than women (OG 1: 19.9%, OG 2: 29.5%, OG 3: 18.7%). In OG 3, 13.2% of women and 13.3% of men stated that they take drugs used for the treatment of bone diseases. Apart from one man in OG 1, none of the participants in OG 1 and OG 2 took drugs of this category. All described differences by OG were statistically significant in both women and men. 

Women (OG 1: 78.0 ± 19.9, OG 2: 85.7 ± 16.1, OG 3: 75.5 ± 21.8) and men (OG 1: 86.0 ± 15.9, OG 2: 90.5 ± 14.2, OG 3: 73.3 ± 29.4) in OG 2 showed the highest mean physical functioning scores (*p* < 0.001). Participants in OG 2 most often reached the norms in the 30 s chair stand test, the 2 min step test, the sit-and-reach test, and the back scratch test (*p* < 0.05 for all listed measurements in women and men). Statistically significant group differences were not observed for handgrip strength in either women or men. 

Table 5 displays the participants’ adherence to lifestyle recommendations for bone health. Independent of sex and osteoporosis status, almost all participants fulfilled the recommendations for calcium intake. Adherence to vitamin D recommendations was lower than for calcium. There were no significant differences by OG in women and men for calcium and vitamin D intake. In regard to sun exposure, almost all participants reached the recommendations. Although still high, the lowest adherence was observed in participants in OG 3 (women: 91.2%, men: 86.7%). Group differences regarding sun exposure were only statistically significant in men (women: *p* = 0.087, men: *p* = 0.034). Overall, alcohol consumption was higher among men compared to women. Similar proportions of women in OG 1 (72.4%) and OG 3 (72.2%) reached the recommendations, while the proportion was slightly lower in women in OG 2 (63.6%). Group differences in women were statistically significant (*p* = 0.025). In men, differences by OG regarding alcohol consumption did not reach statistical significance (*p* = 0.656). The proportion of women who accumulated a minimum of two hours of resistance/weight-bearing exercise per week ranged from 36.5 to 54.4%, with the highest adherence in OG 2 (*p* < 0.001). In men, there were no statistically significant differences by OG regarding resistance/weight-bearing exercise. In general, more women reached the physical activity recommendations of a minimum of 150 min per week. Adherence was highest in OG 2 (57.7%), followed by OG 3 (49.4%) and OG 1 (44.0%). In men, 14.3% in OG 3 fulfilled the recommendations, 30.6% in OG 1, and 45.3% in OG 2. These differences by OG were statistically significant in women (*p* = 0.002) and men (*p* = 0.004).

The number of fulfilled lifestyle recommendations for bone health by sex and osteoporosis status are displayed in Table S1. Table 6 shows the frequencies of participants with lower adherence to lifestyle recommendations for bone health (one to three recommendations fulfilled) dependent on their educational status, socioeconomic status, and living situation. None of these variables showed statistically significant differences by OG.

## 4. Discussion

This study investigated the osteoporosis status of older adults and their adherence to lifestyle recommendations for bone health. Women were more often diagnosed with osteoporosis or were at high risk compared to men. Participants in OG 2 reported the highest self-rated health and reached the best results in the physical fitness measurements. Overall, adherence to recommendations for calcium intake, vitamin D intake, and sun exposure was high. Lower adherence was observed regarding resistance/weight-bearing exercise, physical activity, and alcohol consumption. Female participants had higher frequencies of lower adherence to bone health recommendations with increasing socioeconomic status.

In our study, 10.6% of women and 2.0% of men reported a diagnosis of osteoporosis. Numbers for a direct comparison of osteoporosis prevalence in Germany are not available because either there was no further age categorization for individuals aged 65 years and older [6] or the categories were defined differently [51]. The osteoporosis prevalence in our sample of 65–75-year-old adults is comparable to the estimated prevalence for women and men aged 60–69 years (women: 10.6%, men: 1.7%) by Wade et al. [51]. However, their estimated numbers for 70–79-year-old are considerably higher (women: 23.0%, men: 5.6%) [51]. Osteoporosis is often referred to as a ‘silent disease’ since, in most cases, it remains unrecognized until the first fracture occurs [52,53]. The assessment of osteoporosis through self-report, therefore, underestimates the actual prevalence according to the WHO definition of osteoporosis based on bone mass density [54]. 

We used the OST to classify whether participants are at low or high risk of developing osteoporosis. The OST is based solely on age and body weight, with osteoporosis risk considered higher in individuals with older age and lower weight [37]. Consequently, most participants with normal weight are in OG 2, while overweight or obese participants are more frequently in OG 1. Body mass index is inversely associated with physical functioning [55,56] and physical activity [57] in older adults. This gives an explanation for the better results of physical functioning and fitness among participants in OG 2. The only exception was handgrip strength, where participants of OG 1 most often reached the norm. However, handgrip strength is known to be positively associated with body weight [58].

Most participants were classified to have sufficient vitamin D intake (76.9% to 93.3%) and sun exposure (86.7% to 97.7%). This is contrary to the results of other studies, which reported that only a minority of older adults in Germany reached the reference values for vitamin D intake [30,59]. Given that vitamin D deficiency is widely distributed in Germany [30], our results must be viewed with caution. Assessment was based on self-reported answers of a food frequency questionnaire, which are prone to overestimate dietary consumption [60]. Moreover, the dietary intake was estimated by frequency of consumption alone and did not include size of portions. This is also the case for calcium. Our results suggest that almost all participants have sufficient calcium intake (93.3% to 100.0%). Previous research reported that only 65% of women and 61% of men aged 65–80 years reached the recommended intake levels [59].

Sex differences regarding adherence to recommendations on alcohol consumption were observed. More than half of the male participants consumed alcohol several times per week (women: 27.6% to 36.4%). Patients’ knowledge of the inverse association between alcohol consumption and osteoporosis might be low [25,61]. Further research on this topic is needed to potentially optimize prevention strategies.

In regard to physical activity and resistance/weight-bearing exercise, potential for improvement was observed across all osteoporosis groups. Previous research has shown that older adults tend to engage in physical activities that are easy on the joints (e.g., swimming, cycling) [62], yet these are generally not beneficial for bone health [23]. Moreover, a diagnosis of osteoporosis is often accompanied with insecurities and fear of falling [63], thus leading to a reduction in overall physical activity and, especially, strenuous activities [64]. However, this is not reflected in our study results. 

Even though most participants reached four to six recommendations, only a minority fulfilled all six recommendations. On average, women showed a higher adherence than men, yet the observed differences by sex were small. Other studies reported that sex differences exist regarding osteoporosis knowledge and personal affliction [28,65]. While it is true that women are more often affected by osteoporosis, many men considered the disease as an exclusive women’s problem and were convinced of their good bone health [26,28]. Moreover, men had worse knowledge of osteoporosis than women [28,65]. However, it has also been shown that the majority of women are not ready to perform osteoporosis-preventive behavior [66].

In addition, our results do not indicate that individuals at high risk or with a diagnosis of osteoporosis pay more attention to their lifestyle choices concerning bone health. Individuals at high risk may not be aware of it and individuals with an osteoporosis diagnosis often underestimate the condition [28]. Moreover, osteoporosis is often not recognized as a disease but as an inevitable consequence of ageing [28]. This could decrease the willingness to actively maintain or improve bone health as it has been shown that perceived fracture risk is associated with fracture preventive behavior [67].

Apart from the discussed limitations regarding the classification of osteoporosis risk and the self-assessment of dietary intake, there are further limitations that need to be addressed. Even though smoking is a relevant lifestyle-related risk factor for osteoporosis, smoking status of the participants was not assessed and could, therefore, not be considered in the analyses. Furthermore, the analyses of this study are based on cross-sectional data, which limits the interpretability of the results and does not allow any causality conclusions.

Nevertheless, this study provides a comprehensive picture of osteoporosis prevention behavior among older adults. In this context, the objective assessment of physical activity and physical fitness should be emphasized. 

## 5. Conclusions

In conclusion, findings from this study emphasize the need for tailored bone health-promoting interventions. While adherence to recommendations on dietary intake and sun exposure was high, we observed great potential in bone health-promoting physical activity behavior. Older adults who are already diagnosed with osteoporosis or are at high risk of developing osteoporosis should be specifically addressed by prevention programs.

## Figures and Tables

**Table 1:** Overview of requirements to fulfill lifestyle recommendations for bone health.

	Requirements to Fulfill Recommendations
Calcium intake	Daily consumption of mineral water OR 2 Consumption of a minimum of two calcium-rich foods (hard cheese, soft cheese, milk/buttermilk, yogurt/kefir, cabbage/green vegetables) at least several times a week OR 3 Calcium supplementation
Vitamin D intake	Daily consumption of hard cheese or eggs OR 2 Consumption of hard cheese and eggs at least several times a week OR 3 Consumption of fat fish at least once a week OR 4 Vitamin D supplementation
Sun exposure	At least 20 min per day spent outdoors
Alcohol consumption	Consumption of alcohol a maximum of once per week
Resistance/weight-bearing exercise	Engagement in resistance/weight-bearing exercise (hiking/running, gym training, aerobics, sports therapy, dancing, ball sports) for at least two hours per week
Physical activity	Engagement in at least 150 min of moderate-to-vigorous physical activity per week

**Table 2:** Osteoporosis status of participants by sex.

	Women (*n* = 859)	Men (*n* = 751)	*p*-Value ^#^
	Mean ± SD	Mean ± SD	
OST score *	0.23 ± 2.63	3.25 ± 2.76	**<0.001**
	*n* (%)	*n* (%)	
OG 1	311 (36.2)	425 (56.6)	**<0.001**
OG 2	457 (53.2)	311 (41.4)	
OG 3	91 (10.6)	15 (2.0)	

* Only participants without a diagnosis of osteoporosis. ^#^ *p*-value for statistical significance of sex differences in mean value or frequency distribution; bold numbers indicate statistical significance at *p* < 0.05. OG 1: low risk of osteoporosis. OG 2: high risk of osteoporosis. OG 3: diagnosis of osteoporosis. OST: Osteoporosis Self-Assessment Tool. SD: Standard deviation.

**Table 3:** Characteristics of the participants by sex and osteoporosis status.

	Women (*n* = 859)				Men (*n* = 751)			
	OG 1 (*n* = 311)	OG 2 (*n* = 457)	OG 3 (*n* = 91)	*p*-Value ^#^	OG 1 (*n* = 425)	OG 2 (*n* = 311)	OG 3 (*n* = 15)	*p*-Value ^#^
	*n* (%)	*n* (%)	*n* (%)		*n* (%)	*n* (%)	*n* (%)	
Educational status								
Advanced education (ISCED level ≥ 5)	105 (33.9)	184 (40.3)	31 (34.1)	0.052	262 (62.2)	205 (66.8)	10 (66.7)	0.529
Specialized education (ISCED level 3 + 4)	158 (51.0)	184 (40.3)	41 (45.1)		143 (34.0)	89 (29.0)	4 (26.7)	
Basic education (ISCED level 1 + 2)	47 (15.2)	89 (19.5)	19 (20.9)		16 (3.8)	13 (4.2)	1 (6.7)	
Socioeconomic status								
Upper class	46 (14.8)	97 (21.3)	10 (11.2)	**0.018**	102 (24.1)	88 (28.4)	4 (26.7)	0.561
Middle class	192 (61.9)	277 (60.9)	54 (60.7)		268 (63.2)	182 (58.7)	8 (53.3)	
Lower class	72 (23.2)	81 (17.8)	25 (28.1)		54 (12.7)	40 (12.9)	3 (20.0)	
Living situation								
Not alone	197 (63.5)	277 (60.9)	49 (53.8)	0.246	358 (84.2)	251 (82.0)	13 (86.7)	0.620
Alone	113 (36.5)	178 (39.1)	42 (46.2)		67 (15.8)	55 (18.0)	2 (13.3)	

^#^*p*-value for statistical significance of group differences in frequency distributions; bold numbers indicate statistical significance at *p* < 0.05. OG 1: low risk of osteoporosis. OG 2: high risk of osteoporosis. OG 3: diagnosis of osteoporosis. ISCED: International Standard Classification of Education.

**Table 4:** Health and physical fitness of participants by sex and osteoporosis status.

	Women (*n* = 859)				Men (*n* = 751)			
	OG 1 (*n* = 311)	OG 2 (*n* = 457)	OG 3 (*n* = 91)	*p*-Value ^#^	OG 1 (*n* = 425)	OG 2 (*n* = 311)	OG 3 (*n* = 15)	*p*-Value ^#^
	Mean ± SD	Mean ± SD	Mean ± SD		Mean ± SD	Mean ± SD	Mean ± SD	
Age (in years)	68.9 ± 2.7	70.0 ± 3.0	70.2 ± 3.2	**<0.001**	68.9 ± 2.8	70.5 ± 2.8	68.5 ± 2.3	**<0.001**
Weight (in kg)	82.7 ± 9.6	62.5 ± 6.4	65.7 ± 10.9	**<0.001**	94.0 ± 10.7	74.6 ± 6.1	87.6 ± 15.2	**<0.001**
Height (in cm)	164.9 ± 6.6	161.7 ± 6.1	161.2 ± 6.1	**<0.001**	178.9 ± 6.3	173.4 ± 6.0	179.3 ± 7.9	**<0.001**
Height shrinkage (in cm)	−2.8 ± 2.5	-3.0 ± 2.2	-3.8 ± 2.5	**0.002**	-2.2 ± 1.9	-2.7 ± 2.1	-3.6 ± 3.4	**0.001**
	*n* (%)	*n* (%)	*n* (%)		*n* (%)	*n* (%)	*n* (%)	
Body mass index								
Underweight (<18.5 kg/m^2^)	0 (0.0)	6 (1.3)	3 (3.3)	**<0.001**	0 (0.0)	0 (0.0)	0 (0.0)	**<0.001**
Normal weight (18.5–<25.0 kg/m^2^)	19 (6.1)	296 (64.8)	50 (54.9)		33 (7.8)	167 (53.7)	5 (33.3)	
Overweight (25.0–<30.0 kg/m^2^)	141 (45.3)	150 (32.8)	22 (24.2)		235 (55.3)	139 (44.7)	6 (40.0)	
Obesity (≥30.0 kg/m^2^)	151 (48.6)	5 (1.1)	16 (17.6)		157 (36.9)	5 (1.6)	4 (26.7)	
Self-rated health								
Excellent	5 (1.6)	26 (5.8)	1 (1.1)	**<0.001**	11 (2.6)	17 (5.5)	1 (7.7)	**<0.001**
Very good	56 (18.3)	107 (23.7)	16 (17.6)		100 (23.7)	96 (31.0)	3 (23.1)	
Good	181 (59.2)	271 (60.1)	47 (51.6)		248 (58.8)	169 (54.5)	4 (30.8)	
Less good	58 (19.0)	44 (9.8)	23 (25.3)		58 (13.7)	25 (8.1)	3 (23.1)	
Bad	6 (2.0)	3 (0.7)	4 (4.4)		5 (1.2)	3 (1.0)	2 (15.4)	
Intake of drugs used for the treatment of bone diseases	0 (0.0)	0 (0.0)	12 (13.2)	**<0.001**	1 (0.2)	0 (0.0)	2 (13.3)	**<0.001**
	Mean ± SD	Mean ± SD	Mean ± SD		Mean ± SD	Mean ± SD	Mean ± SD	
Physical functioning	78.0 ± 19.9	85.7 ± 16.1	75.5 ± 21.8	**<0.001**	86.0 ± 15.9	90.5 ± 14.2	73.3 ± 29.4	**<0.001**
	*n* (%)	*n* (%)	*n* (%)		*n* (%)	*n* (%)	*n* (%)	
Handgrip strength (in norm)	241 (78.5)	319 (71.7)	64 (71.1)	**0.088**	322 (77.4)	218 (71.0)	12 (80.0)	**0.131**
30 s chair stand test (in norm)	200 (66.7)	346 (79.4)	61 (71.8)	**0.001**	289 (71.5)	248 (81.8)	5 (38.5)	**<0.001**
2 min step test (in norm)	202 (68.5)	346 (78.8)	60 (70.6)	**0.005**	292 (71.4)	248 (82.1)	9 (64.3)	**0.002**
Sit-and-reach test (in norm)	217 (73.1)	359 (82.3)	65 (78.3)	**0.011**	288 (71.5)	245 (81.4)	6 (54.5)	**0.002**
Back scratch test (in norm)	189 (64.7)	360 (82.9)	65 (74.7)	**<0.001**	287 (71.2)	246 (82.3)	11 (78.6)	**0.002**
4-stage balance test								
1 accomplished	308 (99.7)	452 (98.9)	89 (98.9)	0.433	422 (99.5)	310 (99.7)	14 (100.0)	1.00
2 accomplished	305 (98.7)	449 (98.2)	89 (98.9)	0.917	416 (98.1)	308 (99.0)	14 (100.0)	0.490
3 accomplished	242 (78.3)	377 (82.5)	69 (76.7)	0.225	349 (82.3)	277 (89.1)	12 (85.7)	0.030
4 accomplished	175 (59.6)	311 (68.1)	49 (54.4)	**0.001**	263 (62.0)	236 (75.9)	11 (78.6)	**<0.001**

^#^*p*-value for statistical significance of group differences in mean values or frequency distributions; bold numbers indicate statistical significance at *p* < 0.05. OG 1: low risk of osteoporosis. OG 2: high risk of osteoporosis. OG 3: diagnosis of osteoporosis. SD: Standard deviation.

**Table 5:** Adherence to lifestyle recommendations for bone health of participants by sex and osteoporosis status.

	Women (*n* = 859)				Men (*n* = 751)			
	OG 1 (*n* = 311)	OG 2 (*n* = 457)	OG 3 (*n* = 91)	*p*-Value ^#^	OG 1 (*n* = 425)	OG 2 (*n* = 311)	OG 3 (*n* = 15)	*p*-Value ^#^
	*n* (%)	*n* (%)	*n* (%)		*n* (%)	*n* (%)	*n* (%)	
Calcium intake *	300 (96.8)	441 (97.1)	91 (100.0)	0.234	405 (95.7)	304 (97.7)	14 (93.3)	0.270
Vitamin D intake °	251 (80.7)	353 (77.8)	77 (84.6)	0.274	326 (76.9)	254 (81.7)	14 (93.3)	0.114
Sun exposure (min. 20 min/day)	290 (95.4)	436 (96.5)	83 (91.2)	0.087	414 (97.4)	300 (97.7)	13 (86.7)	**0.034**
Alcohol consumption	223 (72.4)	285 (63.6)	65 (72.2)	**0.025**	203 (48.1)	140 (45.3)	6 (40.0)	0.656
Resistance/weight-bearing exercise (min. 2 h/week)	111 (36.5)	245 (54.4)	39 (43.8)	**<0.001**	153 (36.3)	131 (42.8)	7 (46.7)	0.177
Physical activity (min. 150 min/week)	121 (44.0)	226 (57.7)	39 (49.4)	**0.002**	118 (30.6)	115 (41.4)	2 (14.3)	**0.004**

* Sufficient calcium intake defined as regular consumption of calcium-rich foods or calcium supplements. ° Sufficient vitamin D intake defined as regular consumption of vitamin D-rich foods or vitamin D supplements. ^#^ *p*-value for statistical significance of group differences in frequency distributions; bold numbers indicate statistical significance at *p* < 0.05. OG 1: low risk of osteoporosis. OG 2: high risk of osteoporosis. OG 3: diagnosis of osteoporosis.

**Table 6:** Participants with lower adherence to lifestyle recommendations for bone health dependent on their characteristics by sex and osteoporosis status.

	Women (*n* = 859)				Men (*n* = 751)			
	OG 1 (*n* = 311)	OG 2 (*n* = 457)	OG 3 (*n* = 91)	*p*-Value ^#^	OG 1 (*n* = 425)	OG 2 (*n* = 311)	OG 3 (*n* = 15)	*p*-Value ^#^
	*n* (%)	*n* (%)	*n* (%)		*n* (%)	*n* (%)	*n* (%)	
Educational status								
Advanced education (ISCED level ≥ 5)	28 (26.7)	39 (21.2)	7 (22.6)	0.336	92 (35.1)	63 (30.7)	2 (20.0)	0.169
Specialized education (ISCED level 3 + 4)	33 (20.9)	27 (14.7)	6 (14.6)		47 (32.9)	21 (23.6)	2 (50.0)	
Basic education (ISCED level 1 + 2)	7 (14.9)	15 (16.9)	3 (15.8)		5 (31.2)	4 (30.8)	1 (100.0)	
Socioeconomic status								
Upper class	11 (23.9)	19 (19.6)	2 (20.0)	0.651	33 (32.4)	27 (30.7)	0 (0.0)	0.220
Middle class	43 (22.4)	49 (17.7)	9 (16.7)		90 (33.6)	51 (28.0)	3 (37.5)	
Lower class	15 (20.8)	13 (16.0)	4 (16.0)		22 (40.7)	10 (25.0)	2 (66.7)	
Living situation								
Not alone	42 (21.3)	45 (16.2)	5 (10.2)	0.099	122 (34.1)	67 (26.7)	4 (30.8)	0.414
Alone	27 (23.9)	35 (19.7)	11 (26.2)		24 (35.8)	20 (36.4)	1 (50.0)	

^#^*p*-value for statistical significance of group differences in frequency distributions; bold numbers indicate statistical significance at *p* < 0.05. OG 1: low risk of osteoporosis. OG 2: high risk of osteoporosis. OG 3: diagnosis of osteoporosis. ISCED: International Standard Classification of Education. Lower adherence: one to three recommendations fulfilled.

## Data Availability

The data presented in this study are available on request from the corresponding author. The data are not publicly available due to privacy restrictions.

## Supplementary Materials

The following supporting information can be downloaded at: https://www.mdpi.com/article/10.3390/nu14122463/s1, Table S1: Number of fulfilled lifestyle recommendations for bone health by sex and osteoporosis status.

## Abbreviations

ATC	Anatomical Therapeutic Chemical
BMI	Body mass index
ISCED	International Standard Classification of Education
MVPA	Moderate-to-vigorous physical activity
OA1	OUTDOOR ACTIVE pilot study
OA2	OUTDOOR ACTIVE cluster-randomized controlled trial
OG 1	Low risk of osteoporosis
OG 2	High risk of osteoporosis
OG 3	Diagnosis of osteoporosis
OST	Osteoporosis Self-Assessment Tool
WHO	World Health Organization
