# Supplementary material for: Adherence to Lifestyle Recommendations for Bone Health in Older Adults with and without Osteoporosis: Cross-Sectional Results of the OUTDOOR ACTIVE Study
Source: Nutrients. 2022 Jun 14;14(12):2463. doi: 10.3390/nu14122463 (PMC9228189; doi:10.3390/nu14122463)
Supplement: Supplementary file 1 [file nutrients-14-02463-s001.zip › nutrients-1731609-supplementary.pdf]

**Table S1.** Number of fulfilled lifestyle recommendations for bone health by sex and osteoporosis status.

| | Women (n=859) | | | Men (n=751) | | |
|---|---|---|---|---|---|---|
| | OG 1 (n=311) | OG 2 (n=457) | OG 3 (n=91) | OG 1 (n=425) | OG 2 (n=311) | OG 3 (n=15) |
| | n (%) | n (%) | n (%) | n (%) | n (%) | n (%) |
| Number of fulfilled recommendations | | | | | | |
| 0 | 0 (0.0) | 0 (0.0) | 0 (0.0) | 0 (0.0) | 0 (0.0) | 0 (0.0) |
| 1 | 2 (0.6) | 3 (0.7) | 0 (0.0) | 8 (1.9) | 0 (0.0) | 0 (0.0) |
| 2 | 16 (5.1) | 12 (2.6) | 6 (6.6) | 20 (4.7) | 20 (6.4) | 2 (13.3) |
| 3 | 51 (16.4) | 66 (14.4) | 10 (11.0) | 118 (27.8) | 69 (22.2) | 3 (20.0) |
| 4 | 122 (39.2) | 171 (37.4) | 37 (40.7) | 193 (45.4) | 136 (43.7) | 7 (46.7) |
| 5 | 99 (31.8) | 153 (33.5) | 24 (26.4) | 71 (16.7) | 63 (20.3) | 3 (20.0) |
| 6 | 21 (6.8) | 52 (11.4) | 14 (15.4) | 15 (3.5) | 23 (7.4) | 0 (0.0) |
| | Mean ± SD | Mean ± SD | Mean ± SD | Mean ± SD | Mean ± SD | Mean ± SD |
| Mean number of fulfilled recommendations | 4.17 ± 1.00 | 4.35 ± 0.99 | 4.33 ± 1.08 | 3.81 ± 0.95 | 4.00 ± 0.99 | 3.73 ± 0.96 |

OG 1: low risk of osteoporosis
OG 2: high risk of osteoporosis
OG 3: diagnosis of osteoporosis
SD: Standard deviation
